# Supplementary material for: Exploring the spatial spillover effects of Yangtze River Delta ports on urban economic growth
Source: PLoS One. 2024 Aug 19;19(8):e0307770. doi: 10.1371/journal.pone.0307770 (PMC11332945; doi:10.1371/journal.pone.0307770)
Supplement: Attachment — Submitted filename: Response to reviewers.docx [file pone.0307770.s002.docx]

Ju-Ming Shi

School of Economics and Management

Shanghai Maritime University

Shanghai 201306, P. R. China

*E-mail: shijuming@stu.shmtu.edu.cn*

March 30, 2024

Muhammad Khalid Bashir, PhD
Academic Editor
*PLOS ONE*

Dear professor Bashir

We would like to thank you, the Editor and two anonymous referees for the careful and comprehensive reviews of our manuscript (PONE-D-23-38795) “**Exploring the spatial spillover effects of Yangtze River Delta ports on urban economic growth**”. We also appreciate very much the opportunity that you gave us to revise our manuscript for possible publication by ***PLOS ONE.*** We have made substantial and extensive revisions to the manuscript by addressing all the comments from the review team.

A detailed point to point reply to the reports is given on the following pages. All changes have been incorporated in the revision, following the comments and suggestions.

In addition, in response to the discrepancies noted between the ‘Funding Information’ and ‘Financial Disclosure’ sections, we have included a statement about our funder in the Cover Letter.

Lastly, throughout the course of the manuscript’s revision, Edwin Kuang has made some contributions, including the conceptualization of the literature review and enhancements to the articulation and refinement of the manuscript. Therefore, we are considering adding him as a co-author to the manuscript.

Yours sincerely,

Ju-Ming Shi

**Response to the comments of Referee 1**

Thank you very much for your comments and suggestions. In the following, we copy your comments in italic and follow with our response. We kindly request that reviewers consider our page numbers and line numbers as the reference due to potential inconsistencies with the system's format. We deeply appreciate your understanding of this matter.

**1.** *A literature review was conducted on “the interrelation between port enhancement and regional economic prosperity” and “the interrelation between financial development and economic prosperity”, respectively, but should logically link them together. Meanwhile, “literature review” is not simply a list of literature.*

**Our answer:** In the sections that discuss the impact of ports on urban economic development and the role of financial development in enhancing the contribution of ports to the growth of urban economies, we have conducted a comprehensive literature review, reorganizing the literature in a more logical manner, and linking the concepts together. Furthermore, we have identified the limitations of previous studies and highlighted how our research differs from existing literature. The revised details span from lines 3 on page 6 to 10 on page 10, or are provided below as follows:

“Extensive research confirms that ports enhance the flow of goods and capital, diminish transaction costs, and widen market connectivity [4, 5], yielding significant economic and social benefits for local cities. Specifically, the development of ports can directly influence the growth of related industries, such as loading and unloading, logistics, warehousing, and equipment manufacturing, among other port-related industries. Meanwhile, in cities with more developed financial sectors, the expansion of ports can also lead to the rise of port service industries such as insurance, financial, and commercial services [3, 6].

In reviewing the past studies on the relationship between ports and regional economic growth, this paper finds that scholars have utilized a variety of methods to explore the impact of ports on the hinterland economy. These include traditional input-output analysis [7], grey relational analysis [8], and structural equation modeling [9]. These methods uncover the intricate relationship between port activities and regional economies from diverse angles. However, such methods have limitations and do not consider the spatial agglomeration characteristics of both ports and cities. Nonetheless, as key nodes of urban economic activities, the impact of ports on regional economies does not occur in isolation. Ports may generate spillover effects in the surrounding areas through trade dynamics and market adaptation mechanisms, which can have both positive and negative impacts on the economies of adjacent areas [10]. Furthermore, since port cities are more likely to gain comparative economic advantages, they often become the epicenters of economic agglomeration [11]. Under the influence of agglomeration forces, urban economies may self-reinforce, thereby gaining momentum for long-term economic growth [9]. This agglomeration can self-reinforce urban economies, propelling long-term growth and extending spillover effects to surrounding regions.

In recent years, the topic of whether ports can generate economic spillover effects on surrounding areas has gradually sparked heated discussions, contributing to a rich literature. With the growth of global trade volume, the flow of cargo handled by ports has significantly increased, necessitating the improvement of corresponding transportation infrastructure. Consequently, ports can extend their economic impact to surrounding areas through a widespread transportation network [12]. Clearly, the spillover effects of ports are complex and can have either positive or negative impacts on surrounding areas, leading to no unanimous conclusion. For instance, Bottasso et al., in their study of the impact of Western European ports on regional economies, found using spatial econometric models that an increase in port throughput also raises the local GDP, and has a positive spillover effect on the GDP of neighboring regions, indicating that port activities generate positive externalities [10]. However, negative impacts can also arise. For example, Cohen and Monaco using data from California, USA, found that investment in port infrastructure could promote the development of the state’s retail industry but produced a negative spillover effect on surrounding areas, creating a “siphoning effect” [13].

As a country with an extensive coastline, the construction and development of ports have always been an integral part of China’s economic strategy. Studies on the impact of Chinese ports on regional economic development are widespread, focusing broadly on industrial agglomeration, employment and social development, and regional economic growth and integration, among others. In terms of industrial agglomeration, Xu and Wang found that ports have played a promotional role in the logistics industry agglomeration in the YRD region, and this type of industry agglomeration further promotes regional economic growth [14]. Regarding employment, Wang and Wang believe that port investment stimulates potential demand in upstream and downstream industries, directly and indirectly increasing the output of other industries to promote employment[15]. In terms of regional economic growth, Deng et al. using the spatial Durbin model to study 46 coastal ports in China, found that ports have a significant economic spillover effect on surrounding cities [16].

In the process of reviewing the existing literature, it was found that current research mainly focuses on integrating various port clusters for study, rarely conducting separate analyses. However, considering China’s major port clusters include the Bohai Rim, Yangtze River Delta, Pearl River Delta, Southeast Coast, and Southwest Coast regions, each port cluster’s development level is not the same, and the distances between each port cluster are further compared to the distances within the port clusters themselves. If studied collectively, it might underestimate or overestimate the role of individual port clusters in promoting economic growth in surrounding cities. Therefore, this paper considers focusing the research scope within the YRD port area, making the research conclusions clearer and more comprehensive, and thus the countermeasure suggestions more actionable.

While numerous studies have addressed the spillover effects of ports on regional economies, few have delved into the specifics of these effects’ boundaries. Recognizing that spillover effects diminish with increased spatial distance, and that surrounding cities experience these effects differently, identifying the spatial limits of these effects is crucial. Such insight would enable higher-level governments to craft strategic plans that maximize the beneficial impacts of ports, fostering economic integration and balanced development across regions. Additionally, the variation in development levels between ports, along with the distinct functions served by inland versus coastal ports, results in varied spillover effects. Yet, there appears to be a gap in existing research regarding a detailed analysis of these distinctions. Therefore, this paper seeks to fill this gap by adopting a heterogeneity-focused approach to examine the distinct spillover effects generated by individual ports, distinguishing between inland and coastal ports. This perspective aims to provide a more comprehensive understanding of how different types of ports contribute to regional economic dynamics.

Financial development holds a significant link to the economic dynamics of port areas, serving as a crucial component of the socio-economic wealth in these regions [17]. In the studies related to the relationship between ports and regional economics, some scholars have affirmed that financial development can promote the economic prosperity of port areas. For example, Hesse and Rodrigue highlighted that financial development underpins the construction and operational aspects of logistics infrastructure and goods distribution, further amplifying economic benefits across regions through the transportation network [18]. However, the influence of financial development on the relationship between ports and regional economic growth is not linear; its effects may become ineffective or even produce negative impacts. For instance, Musso et al. examined the contrasting impacts of public and private investments on regional economies linked to ports, noting that private investments might neglect external positives, while public investments could result in the inefficient allocation of financial resources, failing to bolster port-related industry development effectively [19]. Moreover, the relationship between financial development and the regional economy concerning ports is also affected by regional economic structures, policy environments, legal frameworks, and market maturity [20].

Reflecting on the existing literature, found that scholars tend to explore the interaction between financial development and port economies without considering the role played by different levels of financial development in influencing the impact of ports on regional economies. Therefore, how to effectively manage and control financial development to maximize its positive effects in the economic influence of ports on their regions is the focus of this study. To achieve this, the paper employs the threshold effect model to investigate how different financial development stages modify the capacity of ports to spur regional economic growth.”

**2.** *The theoretical value and practical implications of the study are not clearly presented in the introduction or literature review.*

**Our answer:** In the first chapter, specifically in the third and fourth paragraphs, we have outlined the theoretical value and practical implications of our work, respectively. Additionally, the final paragraph of the first chapter enumerates the marginal contributions of our research. The revised details span from lines 18 on page 3 to 18 on page 5, or are provided below as follows:

“The Yangtze River Delta (YRD) region, encompassing Shanghai and select areas of Jiangsu and Zhejiang provinces, is celebrated for its flourishing manufacturing and high-tech industries, alongside a vigorous financial services sector. This makes it one of China’s most advanced and dynamic economic regions, serving as a critical growth pole. Distinguished by its abundant water resources and favorable water transportation conditions, the YRD’s port infrastructure plays a crucial role. Amidst the backdrop of globalization and the international division of labor, the YRD’s sophisticated port facilities have positioned it as an essential node within both China’s and the global trade networks. These ports not only serve as conduits linking domestic and international markets but also act as vital engines propelling regional economic collaborative development, exerting a significant influence on the global trade landscape. The Chinese government’s strategic focus on and enhancement of the YRD integration strategy, particularly through the enactment of the “14th Five-Year Plan for the Integrated Development of the Yangtze River Delta” in 2021, accentuates the pivotal role of these ports in regional economic development. Thus, a comprehensive exploration of the economic impacts of YRD port cities is paramount, enriching the theoretical foundations of economics, geography, and urban planning, while providing empirical support for policy-making, fostering the economic proliferation of port cities, and ensuring the efficient functioning of ports.

The YRD ports are not merely crucial gateways for China’s integration into the global sphere but also primary catalysts for the swift economic development and integration of the region. By facilitating the efficient allocation and circulation of resources, these ports have knit tight economic ties and a robust logistics network, acting as essential hubs for both the aggregation and distribution of goods, as well as pivotal junctions for the flow of information, capital, and talent. As integral components of the industrial chain, YRD ports have forged close upstream and downstream relationships with adjacent manufacturing, service, and various industries, attracting a significant aggregation of production and service enterprises through their logistical and locational benefits. These clusters, through the sharing of resources, technology, and market information, further accelerate the integrated and collaborative development of the regional economy. Studying the spatial spillover effects of YRD ports on urban economic growth offers policymakers a scientific basis for port and regional collaborative development strategies, aiding in the formulation of precise policies aimed at promoting the coordinated and balanced advancement of ports and the regional economy, thereby possessing significant practical value.

In the literature review section, this paper organizes related literature, and compared to current research, presents the marginal contributions of this article: (1) This paper focuses its research scope on the YRD region, not only enabling a deeper analysis of the region’s uniqueness but also proposing more actionable countermeasure suggestions. (2) This paper conducts ordinary regression analysis on the economic development levels of various cities and their geographical distances from Shanghai, preliminarily identifying the spatial connections between port cities. (3) It analyzes how the spatial distance between port cities affects the spillover effects of urban economic growth and determines the geographical decay boundaries of spillover effects. (4) Utilizing a spatial Durbin model with heterogeneity coefficients, it studies the spatial spillover effects of each port city on economic growth. (5) Through heterogeneity analysis, it explores the differences in spillover effects exhibited by coastal port cities and inland port cities in urban economic growth. (6) Using a threshold effect model, it investigates the nonlinear role of financial development in the impact of ports on urban economic growth.”

**3.** *Instead of listing each common models, the research article needs to articulate the applicability and strengths of the chosen one.*

**Our answer:** In the first paragraph of Section 4.4 (Chapter 4), we present the applicability and advantages of our chosen model, contrasting it with the relative shortcomings of alternative models. Furthermore, in Section 5.2.2 (Chapter 5), we provide the rationale behind selecting the spatial Durbin model, including the testing methods and sequence employed. The added details span from line 2 to 15 on page 18, and from line 9 on page 23 to line 4 on page 24, or are provided below as follows:

“New Economic Geography suggests that there is a certain correlation between different geographic locations, which means that when studying the impact of the YRD ports on urban economic growth, it is necessary to consider this spatial interaction. Therefore, spatial econometric models are employed for analysis. Common spatial econometric models include the Spatial Lag Model (SLM), Spatial Error Model (SEM), and Spatial Durbin Model (SDM). Considering this paper aims to study the spatial spillover effects of ports on urban economic growth, with urban economy as the dependent variable and ports as the independent variable. Simply put, SLM and SEM models analyze the spillover effects through the spatial lag of the dependent variable, meaning they look at how urban economies influence each other but cannot determine what causes these spillover effects. The SDM model, on the other hand, not only considers the spatial lag effects of the dependent variable (how the dependent variables of neighboring areas affect the dependent variable of the local area) but also the spatial lag effects of the independent variables (how the independent variables of neighboring areas affect the dependent variable of the local area) and possible spatially correlated errors, making it an extension or combination of the SLM and SEM models.”

“The Moran test indicates that there is a spatial effect between ports and urban economic growth, but relying solely on this to construct a spatial econometric model is not reasonable; other tests are also needed. Following the earlier discussion, using the SDM model for further research is more in line with the theme of this paper, but this requires a series of tests. First, the LM test is used to determine if SEM or SLM models can be applied; if the test fails, spatial econometric methods cannot be used. If the test is successful, then its robustness needs to be tested; if Robust LM test fails, only SEM or SLM models can be adopted. If the test is successful, then LR or Wald tests are used to decide if the SDM model can be applied. The logic behind these two tests is the same, to test whether the SDM can be simplified to SEM or SLM models. If the LR or Wald tests are passed, it proves that the SDM model is stable, and thus can be used for spatial econometric analysis.”

**4.** *What is the reason behind the conclusion of the paper that “the indirect stimulus on the economies of surrounding cities is more pronounced compared to the direct implications on the economies of the host cities”?*

**Our answer:** In the third paragraph of Chapter 8, we discuss the potential reasons behind the conclusions. Additionally, based on this finding, we outline the improvements in our manuscript and propose one of the directions for future research. The added details span from lines 18 on page 38 to 11 on page 39, or are provided below as follows:

“Compared to the direct driving effect of ports on the economic growth of their local cities, the indirect driving effect of ports on the economic growth of surrounding cities is more pronounced. This conclusion was drawn by comparing the direct and indirect effects of ports, and it remains valid under related robustness tests. The economic growth of a city where a port is located is not solely dependent on the port as a growth pole; other growth poles may be more significant. Additionally, this paper selects port throughput as the indicator of port development level, which is overly simplistic and fails to consider the comprehensive impact generated by ports. Ports can produce a significant economic radiative force and driving effect through multiple pathways, such as promoting regional economic integration, industrial diffusion, infrastructure improvement, job creation, attracting external investment, and enhancing competitiveness and openness. Therefore, focusing solely on the role of ports might underestimate their impact on the local economy. Based on these findings, the areas for improvement and future research directions identified in this paper are to find suitable and compelling evaluation indicators to more accurately assess the impact of ports on urban economic growth and spillover effects.”

**5.** *Generally, research articles need to have an “Discussions” section before “Conclusions”, in which they need to go deeper and improve on the previous research. I suggest that you should restructure this article.*

**Our answer:** We have introduced a new Chapter 8 to the paper. This section delves into two principal topics: Firstly, it delineates the main distinctions between our study and other similar research, highlighting the marginal contributions of our work. Secondly, it explores the possible reasons behind the two conclusions of our study, as well as areas that necessitate future improvements. The added details span from line 3 on page 37 to line 9 on page 40, or are provided below as follows:

“The YRD region boasts a significantly higher number of international hub ports compared to other regions, offering unique advantages for rapid economic development and regional integration. This raises the question of the impact these ports have on the regional economy and to what extent. With these considerations in mind, this paper aims to employ an SDM to investigate the impact and spillover effects of ports on regional economies in this area. However, upon reviewing related literature, the study by Deng et al. (2020) was found to be the most similar [16], in which they calculated the composite strength of China’s five major coastal port clusters and explored how the comprehensive strength of ports affects the economic growth of local and surrounding cities, including the YRD region. A closer examination of this article reveals some differences.

The differences are mainly in two aspects: (1) The article did not consider how the distance between cities affects the spillover effect of ports. Essentially, as the distance for transporting products from ports to surrounding areas increases, logistic costs rise, reducing the competitiveness of products in the receiving areas. This leads to a decline in profits and economic growth, thereby weakening the positive radiative effect of ports. Therefore, neglecting distance in studying spillover effects may introduce bias and affect related conclusions. (2) The article also analyzed how urban capital impacts the economy of coastal port areas, noting specifically that an increase in urban capital has a significant direct impact on local economic growth and has a positive spatial spillover effect on the economic growth of surrounding cities. However, this differs from the content this paper intends to study, which considers the role of ports in influencing urban economic growth, as well as the effects of financial development, rather than conducting a spatial regression between financial development and urban economic growth, which overlooks the role of ports. Thus, this paper considers combining the three for analysis. On one hand, this paper establishes a financial development distance weight matrix, taking it into account when analyzing the spillover effect of ports on urban economic growth. On the other hand, this paper uses a threshold effect model to study the role financial development plays at different levels in the impact of ports on urban economic growth.

Reviewing our article reveals two conclusions not previously addressed by prior research. (1) Compared to the direct driving effect of ports on the economic growth of their local cities, the indirect driving effect of ports on the economic growth of surrounding cities is more pronounced. This conclusion was drawn by comparing the direct and indirect effects of ports, and it remains valid under related robustness tests. The economic growth of a city where a port is located is not solely dependent on the port as a growth pole; other growth poles may be more significant. Additionally, this paper selects port throughput as the indicator of port development level, which is overly simplistic and fails to consider the comprehensive impact generated by ports. Ports can produce a significant economic radiative force and driving effect through multiple pathways, such as promoting regional economic integration, industrial diffusion, infrastructure improvement, job creation, attracting external investment, and enhancing competitiveness and openness. Therefore, focusing solely on the role of ports might underestimate their impact on the local economy. Based on these findings, the areas for improvement and future research directions identified in this paper are to find suitable and compelling evaluation indicators to more accurately assess the impact of ports on urban economic growth and spillover effects.

(2) Compared to coastal ports, which do not exhibit a spillover effect on the economies of surrounding cities, inland ports demonstrate a positive spillover effect. This conclusion was derived from dividing the YRD port cluster into coastal and inland ports and conducting group regression analysis. The difference arises because coastal ports have a unique advantage in international trade, with their economic activities spread globally, potentially resulting in weaker economic ties with surrounding regions compared to inland ports. However, extending the perspective globally to study the spillover effects of coastal ports on other countries might yield different conclusions. Since China’s reform and opening, coastal port cities in the YRD have developed rapidly due to their geographical advantages (not implying that all coastal cities are port cities). This has led to highly uneven development among coastal port cities, which could impact the conclusions of this paper. As discussed in the mechanism analysis section, inland ports are more involved in domestic trade and the domestic economic cycle, entailing more domestic distribution and consumption, thus having a comparative advantage in strengthening economic ties with domestic surrounding areas. Moreover, compared to the economic disparities among coastal port cities, the economic development gap among inland port cities is smaller and relatively balanced. This also influences the conclusions of this paper. Based on this analysis, the areas for improvement and future research directions involve finding suitable samples to intricately study the differences and underlying reasons between coastal and inland port cities in driving the economy of surrounding domestic regions.”

**6.** *The writing of this article is not standard, with many grammatical and formatting errors, such as incorrect line numbers. I strongly advise seeking the assistance of a native English speaker or a professional language editor to enhance the clarity and coherence of your paper.*

**Our answer:** In this revision, we sought assistance from a native English speaker to refine the manuscript, enhancing its clarity and fluency.

**Response to the comments of Referee 2**

Thank you very much for your comments and suggestions. In the following, we copy your comments in italic and follow with our response. We kindly request that reviewers consider our page numbers and line numbers as the reference due to potential inconsistencies with the system's format. We deeply appreciate your understanding of this matter.

**1.** *The language and sentence structures of this manuscript are at times incomprehensible. The writing style in general is awkward at too many instances. The paper needs extensive editing.*

**Our answer:** In this revision, we sought assistance from a native English speaker to refine the manuscript, enhancing its clarity and fluency.

**2.** *This is basically repetition of existing work. Similar studies have also been published in other journals. Author(s) should add a separate paragraph (in the Introduction Section) explaining the shortcomings of previous research and how this study is different from existing studies. I mean contribution of this study in existing literature.*

**Our answer:** The marginal contributions of our paper are outlined in the final paragraph of Chapter 1. Additionally, the last two paragraphs of Section 1 in Chapter 2, as well as the final paragraph of Section 2 in the same chapter, discuss the limitations of previous studies and how our work diverges from them. Furthermore, the second paragraph of Chapter 8 highlights the main differences between our research and the most similar studies in the literature. The related details span from line 6-18 on page 5, lines 7 on page 8 to 10 on page 10, and lines 1-17 on page 38, or are provided below as follows:

“In the literature review section, this paper organizes related literature, and compared to current research, presents the marginal contributions of this article: (1) This paper focuses its research scope on the YRD region, not only enabling a deeper analysis of the region’s uniqueness but also proposing more actionable countermeasure suggestions. (2) This paper conducts ordinary regression analysis on the economic development levels of various cities and their geographical distances from Shanghai, preliminarily identifying the spatial connections between port cities. (3) It analyzes how the spatial distance between port cities affects the spillover effects of urban economic growth and determines the geographical decay boundaries of spillover effects. (4) Utilizing a spatial Durbin model with heterogeneity coefficients, it studies the spatial spillover effects of each port city on economic growth. (5) Through heterogeneity analysis, it explores the differences in spillover effects exhibited by coastal port cities and inland port cities in urban economic growth. (6) Using a threshold effect model, it investigates the nonlinear role of financial development in the impact of ports on urban economic growth.”

“In the process of reviewing the existing literature, it was found that current research mainly focuses on integrating various port clusters for study, rarely conducting separate analyses. However, considering China’s major port clusters include the Bohai Rim, Yangtze River Delta, Pearl River Delta, Southeast Coast, and Southwest Coast regions, each port cluster’s development level is not the same, and the distances between each port cluster are further compared to the distances within the port clusters themselves. If studied collectively, it might underestimate or overestimate the role of individual port clusters in promoting economic growth in surrounding cities. Therefore, this paper considers focusing the research scope within the YRD port area, making the research conclusions clearer and more comprehensive, and thus the countermeasure suggestions more actionable.

While numerous studies have addressed the spillover effects of ports on regional economies, few have delved into the specifics of these effects’ boundaries. Recognizing that spillover effects diminish with increased spatial distance, and that surrounding cities experience these effects differently, identifying the spatial limits of these effects is crucial. Such insight would enable higher-level governments to craft strategic plans that maximize the beneficial impacts of ports, fostering economic integration and balanced development across regions. Additionally, the variation in development levels between ports, along with the distinct functions served by inland versus coastal ports, results in varied spillover effects. Yet, there appears to be a gap in existing research regarding a detailed analysis of these distinctions. Therefore, this paper seeks to fill this gap by adopting a heterogeneity-focused approach to examine the distinct spillover effects generated by individual ports, distinguishing between inland and coastal ports. This perspective aims to provide a more comprehensive understanding of how different types of ports contribute to regional economic dynamics.”

“Reflecting on the existing literature, found that scholars tend to explore the interaction between financial development and port economies without considering the role played by different levels of financial development in influencing the impact of ports on regional economies. Therefore, how to effectively manage and control financial development to maximize its positive effects in the economic influence of ports on their regions is the focus of this study. To achieve this, the paper employs the threshold effect model to investigate how different financial development stages modify the capacity of ports to spur regional economic growth.”

“The YRD region boasts a significantly higher number of international hub ports compared to other regions, offering unique advantages for rapid economic development and regional integration. This raises the question of the impact these ports have on the regional economy and to what extent. With these considerations in mind, this paper aims to employ an SDM to investigate the impact and spillover effects of ports on regional economies in this area. However, upon reviewing related literature, the study by Deng et al. (2020) was found to be the most similar [16], in which they calculated the composite strength of China’s five major coastal port clusters and explored how the comprehensive strength of ports affects the economic growth of local and surrounding cities, including the YRD region. A closer examination of this article reveals some differences.

The differences are mainly in two aspects: (1) The article did not consider how the distance between cities affects the spillover effect of ports. Essentially, as the distance for transporting products from ports to surrounding areas increases, logistic costs rise, reducing the competitiveness of products in the receiving areas. This leads to a decline in profits and economic growth, thereby weakening the positive radiative effect of ports. Therefore, neglecting distance in studying spillover effects may introduce bias and affect related conclusions. (2) The article also analyzed how urban capital impacts the economy of coastal port areas, noting specifically that an increase in urban capital has a significant direct impact on local economic growth and has a positive spatial spillover effect on the economic growth of surrounding cities. However, this differs from the content this paper intends to study, which considers the role of ports in influencing urban economic growth, as well as the effects of financial development, rather than conducting a spatial regression between financial development and urban economic growth, which overlooks the role of ports. Thus, this paper considers combining the three for analysis. On one hand, this paper establishes a financial development distance weight matrix, taking it into account when analyzing the spillover effect of ports on urban economic growth. On the other hand, this paper uses a threshold effect model to study the role financial development plays at different levels in the impact of ports on urban economic growth.”

**3.** *I found several inconsistencies in the 2nd section “Literature review and theoretical hypotheses”.*

*Author(s) should discuss/explain hypotheses in the last paragraph of the Introduction Section.*

*Rewrite hypotheses in a scientific way.*

**Our answer:** Chapter 3, which we have dedicated to mechanism analysis correlating with our hypotheses. This chapter aims to explain the rationale behind our hypotheses, specifically elucidating the pathways through which ports impact urban economic growth and the mechanistic role of financial development in this process. The added details span from lines 11 on page 10 to 8 on page 15, or are provided below as follows:

“The YRD port cluster is among China’s paramount port conglomerates, exerting substantial influence on the region’s economy through its geographical advantages. Initially, the port manifests pronounced industrial agglomeration characteristics, adept at engendering agglomeration effects [21]. As focal points for the convergence of goods, capital, information, and technology, ports leverage a fundamental advantage as platforms for the integration of production factors. To capitalize fully on this advantage, related enterprises often cluster in the vicinity, bolstering cooperation and thereby continuously augmenting industrial scale. This process results in economies of scale, diminishing transportation, production, and transaction information costs, and consequently attracting more enterprises, further expanding industrial scale, and better driving economic growth. Additionally, the port’s locational, infrastructural, and logistical service advantages encourage the agglomeration of enterprises from related industries within port areas. This promotes the concentration of labor, production materials, technology, and capital, facilitating the seamless flow of production factors and thereby propelling economic growth.

Secondly, as a core link in the supply chain, ports can significantly strengthen the synergistic effect between upstream and downstream enterprises by improving the efficiency of cargo circulation and reducing transaction costs [22]. This synergy enhances the mobility of production factors, accelerates the speed of social circulation, and thus improves the response speed and market adaptability of the entire supply chain. Specifically, efficient supply chain synergy bolsters the responsiveness of upstream industries to market fluctuations and furnishes downstream industries with effective raw material supply and product distribution channels. This not only promotes closer economic ties between industries but also enhances the overall efficiency of the regional economy by improving supply chain efficiency.

Lastly, ports also promote trade facilitation, reduce import and export costs, attract more international trade and foreign investment, and increase the openness and attractiveness of the regional economy [18]. Infrastructure improvements, such as the construction of port facilities, roads, and railways, not only directly create jobs but also attract more private and public investment, promoting capital formation and accumulation, providing momentum for the sustained growth of the regional economy. Based on the above analysis, this paper proposes Hypothesis 1.

Hypothesis 1: Yangtze River Delta ports can promote local city economic growth.

Ports, serving as convergence points of water and land transportation and the linchpins of regional economic and trade activities, possess distinct network characteristics [23]. By leveraging their network nature, ports can enhance economic and trade exchanges between regions, accelerate the aggregation and diffusion of logistics and economic activities, and promote the flow of production factors and goods between regions, thereby potentially generating positive or negative spatial spillover effects on regional economic growth [24]. On one hand, ports can generate positive spatial spillover effects on the economic growth of surrounding areas by facilitating the smooth flow of production factors and products between regions. Furthermore, as port infrastructure continuously improves, its linking function gradually strengthens, increasing the economic connections between adjacent areas. The increasingly developed transportation conditions enhance the positive spatial spillover effects on the economic growth of surrounding areas. On the other hand, a well-developed port infrastructure network will direct logistic elements towards economically developed regions, thereby maintaining their advantageous position. Before the peripheral lagging regions can effectively enhance their absorption and development capabilities, they are unable to benefit from the positive spillover effects generated by the port logistics industry of the developed regions. Moreover, if the diffusion effects are less than the agglomeration effects, it could result in the lagging regions experiencing negative spillover effects from the developed areas, thereby exacerbating the imbalance in regional economic development.

The spillover effects of ports on the economies of adjacent regions are determined by multiple factors, including the level of infrastructure, the economic development of the region where the port is located, connectivity and radiating capability, as well as the adaptability, development strategies, and learning imitation effects of surrounding areas [25]. Advanced infrastructure and efficient logistics systems allow certain ports to promote inter-regional economic activities, attract international trade and investment, bringing positive economic spillover effects. However, if the region where the port is located is already economically developed, it may exacerbate regional economic imbalances, draw resources away from surrounding underdeveloped areas, and generate negative spillover effects. Moreover, the connectivity and radiating capability of a port determine its sphere of influence, while the development strategies and learning imitation effects of surrounding areas determine their ability to benefit from port development. Therefore, whether a port can generate positive spillover effects on surrounding areas or lead to negative impacts depends on the combined action of these factors. Finally, coastal ports mainly serve international trade, while inland ports are more oriented towards domestic trade. International trade often involves longer-distance goods transportation and more logistics links, meaning that the economic activities of coastal ports are more likely to spread globally, with relatively weak economic ties to surrounding areas. In contrast, inland ports support more domestic economic circulation, involving more domestic distribution and consumption, and therefore have stronger economic interaction and dependence with surrounding areas.

Based on the first law of geography “Everything is related to everything else, but near things are more related than distant things” [26], the impact of local cities on surrounding areas may be limited by spatial distance [27]. The speed and strength of economic spillovers are related to geographical distance, and the increase in spatial distance gradually weakens the intensity of interaction between these spatial units. As the distance increases, the economic spillover effects of ports gradually weaken. For example, increased transportation costs make it more expensive for cities far from ports to obtain raw materials and export products, reducing their competitiveness; the radiating effect of industrial agglomeration makes it difficult for cities far from ports to enjoy the direct spillover effects of port economic activities; the spread of information and technology is also hindered, affecting these cities’ economic innovation and development speed. Therefore, the economic spillover effects of ports on surrounding cities are influenced by distance. Based on this analysis, this paper proposes Hypothesis 2, Hypothesis 3, and Hypothesis 4.

Hypothesis 2: Ports in the Yangtze River Delta will have a positive spillover effect on the economies of nearby regions.

Hypothesis 3: Different ports have varying economic spillover effects on cities, with coastal ports and inland ports having different impacts on surrounding areas.

Hypothesis 4: With the increase in distance between ports and cities, the spillover effect of the Yangtze River Delta ports exhibits decay and boundaries.

Financial development enhances the depth and efficiency of capital markets, promoting the effective accumulation and allocation of capital. This provides the necessary financial support for the construction of port infrastructure and the expansion of related industries [28], thereby enhancing the throughput capacity and operational efficiency of ports, which helps to drive regional economic growth. However, financial development can also exacerbate the misallocation of resources in ports and related industries. Considering that financial institutions might favor investing in projects with high short-term returns while neglecting the importance of long-term infrastructure construction and maintenance, this could affect the long-term competitiveness of ports and the stability of regional economies [19]. Additionally, financial intermediaries, in providing funding and risk management services, may experience economies of scale and scope. Below a certain scale, the marginal cost of financial intermediation is high, but beyond a critical point, an increase in scale can significantly reduce costs, playing a facilitating role in the process of ports promoting regional economic growth. However, when the scale of financial intermediation becomes too large, problems such as increased management complexity and exacerbated information asymmetry may lead to decreased efficiency, thereby affecting the role of ports in promoting regional economies to some extent. Based on this analysis, this paper proposes Hypothesis 5.

Hypothesis 5: In the impact of ports on urban economic growth, financial development plays a role and exhibits a threshold effect.”

**4.** *Similarly, writing style in the Literature Review section is awkward. Author(s) should add a separate section only on literature review: provide the comments of the cited papers after introducing each relevant work. What readers require is, by convinced literature review, to understand the clear thinking/consideration why the proposed approach can reach more convinced results. This is the very contribution from authors. In addition, authors also should provide more sufficient critical literature review to indicate the drawbacks of existed approaches, then, well define the main stream of research direction, how did those previous studies perform? Employ which methodologies? Which problem still requires to be solved? Why is the proposed approach suitable to be used to solve the critical problem? We need more convinced literature reviews to indicate clearly the state-of-the-art development.*

In Chapter 2, we have established a dedicated section for the literature review and conducted an extensive review of the relevant literature about the impact of ports on urban economic development and the role of financial development in enhancing the contribution of ports to the growth of urban economies. Additionally, in the final two paragraphs of Section 1 in Chapter 2 we highlight the limitations of previous studies and outline the improvements or contributions our paper makes in this context. the marginal contributions of our study are presented in the last paragraph of Chapter 1. The related details span from lines 1 on page 6 to 10 on page 10, and line 6-18 on page 5, or are provided below as follows:

“Extensive research confirms that ports enhance the flow of goods and capital, diminish transaction costs, and widen market connectivity [4, 5], yielding significant economic and social benefits for local cities. Specifically, the development of ports can directly influence the growth of related industries, such as loading and unloading, logistics, warehousing, and equipment manufacturing, among other port-related industries. Meanwhile, in cities with more developed financial sectors, the expansion of ports can also lead to the rise of port service industries such as insurance, financial, and commercial services [3, 6].

In reviewing the past studies on the relationship between ports and regional economic growth, this paper finds that scholars have utilized a variety of methods to explore the impact of ports on the hinterland economy. These include traditional input-output analysis [7], grey relational analysis [8], and structural equation modeling [9]. These methods uncover the intricate relationship between port activities and regional economies from diverse angles. However, such methods have limitations and do not consider the spatial agglomeration characteristics of both ports and cities. Nonetheless, as key nodes of urban economic activities, the impact of ports on regional economies does not occur in isolation. Ports may generate spillover effects in the surrounding areas through trade dynamics and market adaptation mechanisms, which can have both positive and negative impacts on the economies of adjacent areas [10]. Furthermore, since port cities are more likely to gain comparative economic advantages, they often become the epicenters of economic agglomeration [11]. Under the influence of agglomeration forces, urban economies may self-reinforce, thereby gaining momentum for long-term economic growth [9]. This agglomeration can self-reinforce urban economies, propelling long-term growth and extending spillover effects to surrounding regions.

In recent years, the topic of whether ports can generate economic spillover effects on surrounding areas has gradually sparked heated discussions, contributing to a rich literature. With the growth of global trade volume, the flow of cargo handled by ports has significantly increased, necessitating the improvement of corresponding transportation infrastructure. Consequently, ports can extend their economic impact to surrounding areas through a widespread transportation network [12]. Clearly, the spillover effects of ports are complex and can have either positive or negative impacts on surrounding areas, leading to no unanimous conclusion. For instance, Bottasso et al., in their study of the impact of Western European ports on regional economies, found using spatial econometric models that an increase in port throughput also raises the local GDP, and has a positive spillover effect on the GDP of neighboring regions, indicating that port activities generate positive externalities [10]. However, negative impacts can also arise. For example, Cohen and Monaco using data from California, USA, found that investment in port infrastructure could promote the development of the state’s retail industry but produced a negative spillover effect on surrounding areas, creating a “siphoning effect” [13].

As a country with an extensive coastline, the construction and development of ports have always been an integral part of China’s economic strategy. Studies on the impact of Chinese ports on regional economic development are widespread, focusing broadly on industrial agglomeration, employment and social development, and regional economic growth and integration, among others. In terms of industrial agglomeration, Xu and Wang found that ports have played a promotional role in the logistics industry agglomeration in the YRD region, and this type of industry agglomeration further promotes regional economic growth [14]. Regarding employment, Wang and Wang believe that port investment stimulates potential demand in upstream and downstream industries, directly and indirectly increasing the output of other industries to promote employment[15]. In terms of regional economic growth, Deng et al. using the spatial Durbin model to study 46 coastal ports in China, found that ports have a significant economic spillover effect on surrounding cities [16].

In the process of reviewing the existing literature, it was found that current research mainly focuses on integrating various port clusters for study, rarely conducting separate analyses. However, considering China’s major port clusters include the Bohai Rim, Yangtze River Delta, Pearl River Delta, Southeast Coast, and Southwest Coast regions, each port cluster’s development level is not the same, and the distances between each port cluster are further compared to the distances within the port clusters themselves. If studied collectively, it might underestimate or overestimate the role of individual port clusters in promoting economic growth in surrounding cities. Therefore, this paper considers focusing the research scope within the YRD port area, making the research conclusions clearer and more comprehensive, and thus the countermeasure suggestions more actionable.

While numerous studies have addressed the spillover effects of ports on regional economies, few have delved into the specifics of these effects’ boundaries. Recognizing that spillover effects diminish with increased spatial distance, and that surrounding cities experience these effects differently, identifying the spatial limits of these effects is crucial. Such insight would enable higher-level governments to craft strategic plans that maximize the beneficial impacts of ports, fostering economic integration and balanced development across regions. Additionally, the variation in development levels between ports, along with the distinct functions served by inland versus coastal ports, results in varied spillover effects. Yet, there appears to be a gap in existing research regarding a detailed analysis of these distinctions. Therefore, this paper seeks to fill this gap by adopting a heterogeneity-focused approach to examine the distinct spillover effects generated by individual ports, distinguishing between inland and coastal ports. This perspective aims to provide a more comprehensive understanding of how different types of ports contribute to regional economic dynamics.

2.2. The role of financial development in the impact of ports on regional economies

Financial development holds a significant link to the economic dynamics of port areas, serving as a crucial component of the socio-economic wealth in these regions [17]. In the studies related to the relationship between ports and regional economics, some scholars have affirmed that financial development can promote the economic prosperity of port areas. For example, Hesse and Rodrigue highlighted that financial development underpins the construction and operational aspects of logistics infrastructure and goods distribution, further amplifying economic benefits across regions through the transportation network [18]. However, the influence of financial development on the relationship between ports and regional economic growth is not linear; its effects may become ineffective or even produce negative impacts. For instance, Musso et al. examined the contrasting impacts of public and private investments on regional economies linked to ports, noting that private investments might neglect external positives, while public investments could result in the inefficient allocation of financial resources, failing to bolster port-related industry development effectively [19]. Moreover, the relationship between financial development and the regional economy concerning ports is also affected by regional economic structures, policy environments, legal frameworks, and market maturity [20].

Reflecting on the existing literature, found that scholars tend to explore the interaction between financial development and port economies without considering the role played by different levels of financial development in influencing the impact of ports on regional economies. Therefore, how to effectively manage and control financial development to maximize its positive effects in the economic influence of ports on their regions is the focus of this study. To achieve this, the paper employs the threshold effect model to investigate how different financial development stages modify the capacity of ports to spur regional economic growth.”

“In the literature review section, this paper organizes related literature, and compared to current research, presents the marginal contributions of this article: (1) This paper focuses its research scope on the YRD region, not only enabling a deeper analysis of the region’s uniqueness but also proposing more actionable countermeasure suggestions. (2) This paper conducts ordinary regression analysis on the economic development levels of various cities and their geographical distances from Shanghai, preliminarily identifying the spatial connections between port cities. (3) It analyzes how the spatial distance between port cities affects the spillover effects of urban economic growth and determines the geographical decay boundaries of spillover effects. (4) Utilizing a spatial Durbin model with heterogeneity coefficients, it studies the spatial spillover effects of each port city on economic growth. (5) Through heterogeneity analysis, it explores the differences in spillover effects exhibited by coastal port cities and inland port cities in urban economic growth. (6) Using a threshold effect model, it investigates the nonlinear role of financial development in the impact of ports on urban economic growth.”

“In the literature review section, this paper organizes related literature, and compared to current research, presents the marginal contributions of this article: (1) This paper focuses its research scope on the YRD region, not only enabling a deeper analysis of the region’s uniqueness but also proposing more actionable countermeasure suggestions. (2) This paper conducts ordinary regression analysis on the economic development levels of various cities and their geographical distances from Shanghai, preliminarily identifying the spatial connections between port cities. (3) It analyzes how the spatial distance between port cities affects the spillover effects of urban economic growth and determines the geographical decay boundaries of spillover effects. (4) Utilizing a spatial Durbin model with heterogeneity coefficients, it studies the spatial spillover effects of each port city on economic growth. (5) Through heterogeneity analysis, it explores the differences in spillover effects exhibited by coastal port cities and inland port cities in urban economic growth. (6) Using a threshold effect model, it investigates the nonlinear role of financial development in the impact of ports on urban economic growth.”

**5.** *The models used in the analyses are appropriate.*

**Our answer:** In the first paragraph of Section 4.4 (Chapter 4), we present the applicability and advantages of our chosen model, contrasting it with the relative shortcomings of alternative models. Furthermore, in the first paragraph of Section 5.2.2 (Chapter 5), we provide the rationale behind selecting the spatial Durbin model, including the testing methods and sequence employed. The added details span from line 2 to 15 on page 18, and lines 9 on page 23 to 4 on page 24, or are provided below as follows:

“New Economic Geography suggests that there is a certain correlation between different geographic locations, which means that when studying the impact of the YRD ports on urban economic growth, it is necessary to consider this spatial interaction. Therefore, spatial econometric models are employed for analysis. Common spatial econometric models include the Spatial Lag Model (SLM), Spatial Error Model (SEM), and Spatial Durbin Model (SDM). Considering this paper aims to study the spatial spillover effects of ports on urban economic growth, with urban economy as the dependent variable and ports as the independent variable. Simply put, SLM and SEM models analyze the spillover effects through the spatial lag of the dependent variable, meaning they look at how urban economies influence each other but cannot determine what causes these spillover effects. The SDM model, on the other hand, not only considers the spatial lag effects of the dependent variable (how the dependent variables of neighboring areas affect the dependent variable of the local area) but also the spatial lag effects of the independent variables (how the independent variables of neighboring areas affect the dependent variable of the local area) and possible spatially correlated errors, making it an extension or combination of the SLM and SEM models.”

“The Moran test indicates that there is a spatial effect between ports and urban economic growth, but relying solely on this to construct a spatial econometric model is not reasonable; other tests are also needed. Following the earlier discussion, using the SDM model for further research is more in line with the theme of this paper, but this requires a series of tests. First, the LM test is used to determine if SEM or SLM models can be applied; if the test fails, spatial econometric methods cannot be used. If the test is successful, then its robustness needs to be tested; if Robust LM test fails, only SEM or SLM models can be adopted. If the test is successful, then LR or Wald tests are used to decide if the SDM model can be applied. The logic behind these two tests is the same, to test whether the SDM can be simplified to SEM or SLM models. If the LR or Wald tests are passed, it proves that the SDM model is stable, and thus can be used for spatial econometric analysis.”

**6.** *The Discussion Section is missing. Author(s) should add the Discussion Section and that must have a convincing and strong literature to support the findings and unfortunately the writing style in general in the results section is awkward.*

**Our answer:** In Chapter 8, we have introduced a discussion section that elaborates on the improvements our study offers over existing literature and propose one of the directions for future research. This section also delineates the main distinctions between our research and the most closely related studies in the field. Moreover, the first paragraph of Chapter 9 cites conclusions from similar studies to further validate our results. The related details span from lines 3 on page 37 to 9 on page 40, and lines 11 on page 40 to 12 on page 41, or are provided below as follows:

“The YRD region boasts a significantly higher number of international hub ports compared to other regions, offering unique advantages for rapid economic development and regional integration. This raises the question of the impact these ports have on the regional economy and to what extent. With these considerations in mind, this paper aims to employ an SDM to investigate the impact and spillover effects of ports on regional economies in this area. However, upon reviewing related literature, the study by Deng et al. (2020) was found to be the most similar [16], in which they calculated the composite strength of China’s five major coastal port clusters and explored how the comprehensive strength of ports affects the economic growth of local and surrounding cities, including the YRD region. A closer examination of this article reveals some differences.

The differences are mainly in two aspects: (1) The article did not consider how the distance between cities affects the spillover effect of ports. Essentially, as the distance for transporting products from ports to surrounding areas increases, logistic costs rise, reducing the competitiveness of products in the receiving areas. This leads to a decline in profits and economic growth, thereby weakening the positive radiative effect of ports. Therefore, neglecting distance in studying spillover effects may introduce bias and affect related conclusions. (2) The article also analyzed how urban capital impacts the economy of coastal port areas, noting specifically that an increase in urban capital has a significant direct impact on local economic growth and has a positive spatial spillover effect on the economic growth of surrounding cities. However, this differs from the content this paper intends to study, which considers the role of ports in influencing urban economic growth, as well as the effects of financial development, rather than conducting a spatial regression between financial development and urban economic growth, which overlooks the role of ports. Thus, this paper considers combining the three for analysis. On one hand, this paper establishes a financial development distance weight matrix, taking it into account when analyzing the spillover effect of ports on urban economic growth. On the other hand, this paper uses a threshold effect model to study the role financial development plays at different levels in the impact of ports on urban economic growth.

Reviewing our article reveals two conclusions not previously addressed by prior research. (1) Compared to the direct driving effect of ports on the economic growth of their local cities, the indirect driving effect of ports on the economic growth of surrounding cities is more pronounced. This conclusion was drawn by comparing the direct and indirect effects of ports, and it remains valid under related robustness tests. The economic growth of a city where a port is located is not solely dependent on the port as a growth pole; other growth poles may be more significant. Additionally, this paper selects port throughput as the indicator of port development level, which is overly simplistic and fails to consider the comprehensive impact generated by ports. Ports can produce a significant economic radiative force and driving effect through multiple pathways, such as promoting regional economic integration, industrial diffusion, infrastructure improvement, job creation, attracting external investment, and enhancing competitiveness and openness. Therefore, focusing solely on the role of ports might underestimate their impact on the local economy. Based on these findings, the areas for improvement and future research directions identified in this paper are to find suitable and compelling evaluation indicators to more accurately assess the impact of ports on urban economic growth and spillover effects.

(2) Compared to coastal ports, which do not exhibit a spillover effect on the economies of surrounding cities, inland ports demonstrate a positive spillover effect. This conclusion was derived from dividing the YRD port cluster into coastal and inland ports and conducting group regression analysis. The difference arises because coastal ports have a unique advantage in international trade, with their economic activities spread globally, potentially resulting in weaker economic ties with surrounding regions compared to inland ports. However, extending the perspective globally to study the spillover effects of coastal ports on other countries might yield different conclusions. Since China’s reform and opening, coastal port cities in the YRD have developed rapidly due to their geographical advantages (not implying that all coastal cities are port cities). This has led to highly uneven development among coastal port cities, which could impact the conclusions of this paper. As discussed in the mechanism analysis section, inland ports are more involved in domestic trade and the domestic economic cycle, entailing more domestic distribution and consumption, thus having a comparative advantage in strengthening economic ties with domestic surrounding areas. Moreover, compared to the economic disparities among coastal port cities, the economic development gap among inland port cities is smaller and relatively balanced. This also influences the conclusions of this paper. Based on this analysis, the areas for improvement and future research directions involve finding suitable samples to intricately study the differences and underlying reasons between coastal and inland port cities in driving the economy of surrounding domestic regions.”

“To explore the impact of the YRD ports on the region’s economy, this paper first conducts basic regression with port throughput, the distance of each city from Shanghai, and the level of urban economic development. This demonstrates that port throughput influences urban economic development and that there exists a spatial connection between the distance of cities and urban economic development. In the second step, spatial econometric testing methods are employed to test if the data can analyze the spatial lag of independent variables using the spatial Durbin model. This proves the rationality of the chosen model and that ports have a positive impact on the local and surrounding regional economy. It also finds that, compared to the direct driving effect on local urban economic growth, the YRD ports have a more significant indirect driving effect on the economic growth of surrounding cities, like the study conducted by Munim and Schramm [22]. The third step examines how different distances between cities affect the economic spillover effect of ports, finding that the positive spillover effect of ports fluctuates mildly between 55-95km and then drops sharply after 95km until it disappears at 110km, aligning with the findings of Yudhistira and Sofiyandi study [34]. The fourth step constructs a financial development distance matrix and threshold effect model, incorporating the impact of financial development in assessing the influence of ports on urban economies. It finds that financial development has a positive driving effect with a clear threshold effect, Similar to the study conducted by Hesse and Rodrigue [18]. Finally, by dividing the YRD ports into coastal and inland ports and conducting spatial regression in groups, it is found that they exhibit different spillover effects on surrounding areas, with inland ports showing a positive spillover and coastal ports showing none, aligning with the findings of Han et al. [23]. Further findings indicate that individual ports have varying spillover effects on their surroundings, but those with positive spillover effects are mostly inland ports.”

**7.** *The conclusion needs revision. Authors should write what they have actually concluded based on the study’s findings. It should not be the repetition of the abstract.*

**Our answer:** In the first paragraph of Chapter 9, we have rewritten the conclusion to align with the logical flow of the article’s argumentation, ensuring it is not merely a repetition of the abstract. The revised details span from lines 11 on page 40 to 12 on page 41, or are provided below as follows:

“To explore the impact of the YRD ports on the region’s economy, this paper first conducts basic regression with port throughput, the distance of each city from Shanghai, and the level of urban economic development. This demonstrates that port throughput influences urban economic development and that there exists a spatial connection between the distance of cities and urban economic development. In the second step, spatial econometric testing methods are employed to test if the data can analyze the spatial lag of independent variables using the spatial Durbin model. This proves the rationality of the chosen model and that ports have a positive impact on the local and surrounding regional economy. It also finds that, compared to the direct driving effect on local urban economic growth, the YRD ports have a more significant indirect driving effect on the economic growth of surrounding cities, like the study conducted by Munim and Schramm [22]. The third step examines how different distances between cities affect the economic spillover effect of ports, finding that the positive spillover effect of ports fluctuates mildly between 55-95km and then drops sharply after 95km until it disappears at 110km, aligning with the findings of Yudhistira and Sofiyandi study [34]. The fourth step constructs a financial development distance matrix and threshold effect model, incorporating the impact of financial development in assessing the influence of ports on urban economies. It finds that financial development has a positive driving effect with a clear threshold effect, Similar to the study conducted by Hesse and Rodrigue [18]. Finally, by dividing the YRD ports into coastal and inland ports and conducting spatial regression in groups, it is found that they exhibit different spillover effects on surrounding areas, with inland ports showing a positive spillover and coastal ports showing none, aligning with the findings of Han et al. [23]. Further findings indicate that individual ports have varying spillover effects on their surroundings, but those with positive spillover effects are mostly inland ports.”
